# Berberine inhibits high fat diet-associated colorectal cancer through modulation of the gut microbiota-mediated lysophosphatidylcholine

**DOI:** 10.7150/ijbs.81824

**Published:** 2023-04-09

**Authors:** Haitao Chen, Chenxiao Ye, Changhong Wu, Jiali Zhang, Lu Xu, Xuanying Wang, Chao Xu, Jin Zhang, Yong Guo, Qinghua Yao

**Affiliations:** 1 Department of Integrated Chinese and Western Medicine, The Cancer Hospital of the University of Chinese Academy of Sciences (Zhejiang Cancer Hospital), Institute of Basic Medicine and Cancer (IBMC), Chinese Academy of Sciences, Hangzhou, Zhejiang 310022, China.; 2 Integrated Traditional Chinese and Western Medicine Oncology Laboratory, Key Laboratory of Traditional Chinese Medicine of Zhejiang Province, Hangzhou, Zhejiang, 310022, China.; 3 Zhejiang Chinese Medical University, Hangzhou, Zhejiang, 310053, China.; 4 Dr. Neher's Biophysics Laboratory for Innovative Drug Discovery, State Key Laboratory of Quality Research in Chinese Medicine, Macau, 999078, China.; 5 Department of Traditional Chinese Medicine, The Second Hospital Affiliated to Air Force Medical University, Xi'an 710038, China.; 6 Department of Oncology, The First Affiliated Hospital of Zhejiang Chinese Medical University, Hangzhou, Zhejiang, 310003, China.; 7 Key Laboratory of Head & Neck Cancer Translational Research of Zhejiang Province, Hangzhou, Zhejiang, 310022, China.

**Keywords:** Berberine, Western diet, Colorectal cancer, Microbiome, Glycerophospholipid metabolism, Wnt pathway

## Abstract

Dietary fat intake is positively associated with elevated risk of colorectal cancer (CRC). Currently, clinical treatments remian inadequate bacause of the complex pathogenesis of CRC induced by a high-fat diet (HFD). Mechanistically, imbalances in gut microbiota are associated with HFD-associated colorectal tumourigenesis. Therefore, we investigated the anti-tumor activity of berberine (BBR) in modulating the dysregulated gut microbiota and related metabolites by preforming 16S rDNA sequencing and liquid chromatography/mass spectrometry. As expected, BBR treatment significantly decreased the number of colonic polyps, ameliorated gut barrier disruption, and inhibited colon inflammation and related oncogenic pathways in AOM/DSS-induced CRC model mice fed with an HFD. Furthermore, BBR alleviated gut microbiota dysbiosis and increased the abundance of beneficial gut microorganisms, including *Akkermansia* and *Parabacteroides*, in HFD-fed CRC mice. In addition, metabolomics analysis demonstrated significantly altered the glycerophospholipid metabolism during the progression of HFD-associated CRC in mice, whereas BBR treatment reverted these changes in glycerophospholipid metabolites, particularly lysophosphatidylcholine (LPC), which was confirmed to promote CRC cell proliferation and ameliorate cell junction impairment. Notably, BBR had no clear anti-tumor effects on HFD-fed CRC model mice with gut microbiota depletion, whereas transplantation of BBR-treated gut microbiota to gut microbiota-depleted CRC mice recapitulated the inhibitory effects of BBR on colorectal tumourigenesis and LPC levels. This study demonstrated that BBR inhibited HFD-associated CRC directly through modulating gut microbiota-regulated LPC levels, thereby providing a promising microbiota-modulating therapeutic strategy for the clinical prevention and treatment of Western diet-associated CRC.

## Introduction

Colorectal cancer (CRC), as a common gastrointestinal cancer, has a relatively high morbidity and mortality among malignant tumors, and severely endangers human health^1^. Diet plays a well-known and important role in regulating CRC occurrence and development^2^, ^3^. Clinical trials have indicated a significant positive association between high-fat diet (HFD) consumption and CRC risk^4^, ^5^, and studies have confirmed that an HFD promotes colorectal tumourigenesis through modulating the gut microbiota and lipid metabolism^6^. With improvements in lifestyle and socioeconomic status, excess dietary fat intake has become a common dietary pattern. Thus, the prevention and treatment of HFD-associated colorectal tumourigenesis warrant in-depth research.

Berberine (BBR), a natural isoquinoline alkaloid extracted from *Coptis coptidis*, is widely used in the clinical treatment of infectious intestinal diseases in China^7^. Recently, several studies have shown that BBR regulates the microbiota structure, thus alleviating type 2 diabetes, DSS-induced colitis, rheumatoid arthritis, and other conditions^8^-^10^. BBR has also been demonstrated to modulate lipid metabolism disturbances in mice with HFD-induced obesity^11^. Our previous study has indicated that BBR suppressed the inflammatory response via regulating the gut microbiota in AOM/DSS-induced CRC mice fed with a chow diet^12^. Additionally, Wang et al. have reported that BBR alleviated the development of CRC in Apc min/+ mice fed with an HFD and ameliorated perturbations in the enteric microbiome community caused by an HFD^13^. However, the effects of BBR on HFD-associated colorectal tumourigenesis have not been sufficiently studied, and whether the gut microbiota and its metabolites might alter the potential effects of BBR remains largely unclear.

Here, we hypothesized that BBR inhibited HFD-associated colorectal cancer directly through modulation of the gut microbiota and related metabolites. To test our hypothesis, we used AOM/DSS-treated Balb/c mice to confirm the inhibitory effect of BBR on HFD-associated colorectal tumourigenesis. We also performed 16S rRNA sequencing and liquid chromatography/mass spectrometry (LC-MS) to determine the regulatory effects of BBR on the gut microbiota and metabolites in HFD-fed CRC model mice. Subsquently, we determined BBR's effects on regulating the growth of colon cancer cells in vitro. Furthermore, gut microbiome-depleted model mice and fecal microbiota transplantation (FMT) experiment were conducted to verify the crucial roles of the gut microbiota in BBR treatment against HFD-associated colorectal tumourigenesis.

## Materials and Methods

### Conventional AOM/DSS Induced CRC Mouse Models

**Figure 1:**
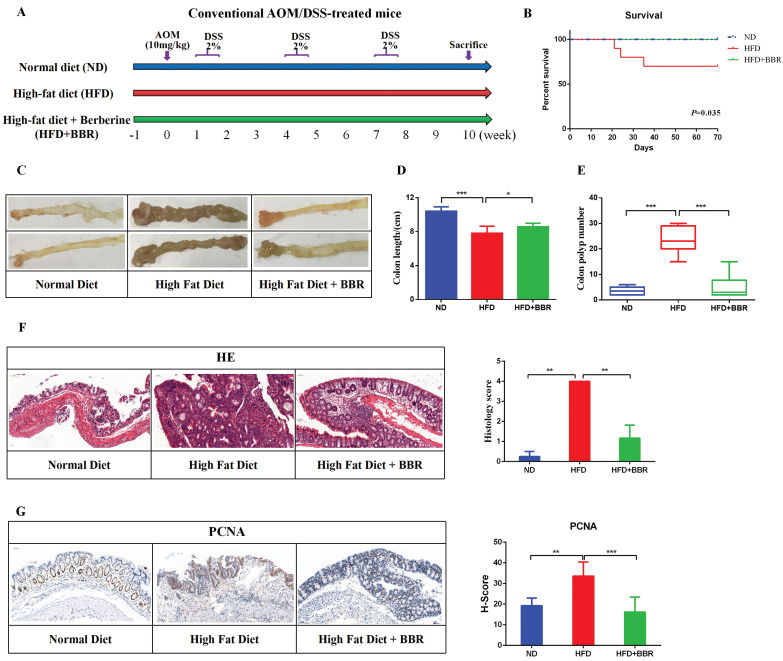
BBR inhibits HFD-associated colorectal tumourigenesis in mice. (A) Design of the BBR experiment in AOM/DSS mice fed with HFD. (B) Survival rates of the three groups. (C) Representative images of the colon at sacrifice. (D) Colon length. (E) Number of colon polyp. (F) HE staining of intestinal sections and the histology score: bar=50μm, (10μm × 5). (G) Immunohistochemistry (IHC) staining of colorectal sections, showing the expression of PCNA: bar=50μm, (10μm × 5). (*p < 0.05, ^**^p < 0.001, ^***^p < 0.001).

Male BALB/c mice at 8 weeks of age were purchased from Shanghai B&K Co., Ltd. (Shanghai, China). All mice were housed in the Laboratory Animal Center of Zhejiang Traditional Chinese Medical University in a controlled environment (20 ± 2℃ with a humidity of 50 ± 5% in a 12 h light/dark cycle with a normal diet (ND) or HFD). After adapting to the diet for 7 days, the mice were randomly divided into three groups (10 mice per group): an ND group, HFD group, and HFD + BBR group. All mice received a single intraperitoneal (i.p.) injection of AOM (10mg/kg, Sigma-Aldrich, USA), followed by three cycles of DSS (2%, MP Bio, USA) administered in the drinking water for 7 days and normal drinking water for 14 days. Meanwhile, HFD+BBR group mice were treated with BBR (100 mg/kg, Shanghai Yuanye Bio-Technology Co., Ltd., China) by oral gavage once daily for 10 weeks. ND group and HFD group mice were gavaged with drinking water (Figure 1A).

To determine the gut microbiota involved in BBR's inhibition of HFD related colorectal tumourigenesis. We adapted male BALB/c mice to the HFD for 7 days, then randomly divided into three groups (10 mice per group): HFD group, HFD+Antibiotic (HFD+ABX) group, and HFD +ABX+BBR group. All mice were administered AOM and DSS according to the previous method. HFD+ABX and HFD+ABX+BBR group mice were given drinking water with an antibiotics cocktail (0.2 g/L ampicillin, neomycin and metronidazole, and 0.1 g/L vancomycin) to deplete the gut microbiota for 2 weeks before the experiment, and every other 2 weeks throughout the entire experiment, as previously reported^6^. HFD+ABX+BBR group mice were treated with BBR (100 mg/kg) by oral gavage once daily for 10 weeks. HFD group and HFD+ABX group mice were gavaged with drinking water (Figure 8A).

### Fecal Microbiota Transplantation (FMT) Experiment

To determine the direct contribution of BBR regulated-gut microbiota to the effects aganist HFD-related colorectal tumourigenesis, we treated male BALB/c mice at 8 weeks of age with an antibiotics cocktail for 2 consecutive weeks before FMT, then divided the mice into 2 groups (8 mice per group) fed with ND, and gavaged with fecal samples from HFD-fed AOM/DSS model mice or HFD+BBR fed AOM/DSS model mice. Briefly, 200 mg of stool samples was homogenized in 1mL of PBS. Then recipient mice were administered 0.2mL of the suspension by gastric gavage for twice per week. All mice also received AOM and DSS to induce colorectal neoplasia. Mice were sacrificed at week 18 (Figure 8A).

### Western blot Analysis

Western blot analysis was performed as described previously^14^. Briefly, colon or cell proteins were subjected to SDS-PAGE and transferred to PVDF membranes, which were sliced for concurrent detection with different primary antibodies. β-actin (1:1000, catalog no. 66009-1-Ig, Proteintech, USA), Occludin (1:1000, catalog no. ab216327, Abcam, UK), ZO-1 (1:1000, catalog no. ab216880, Abcam, UK), PCNA (1:500, catalog no. AF0239, Affinity, USA), IL-1β (1:1000, catalog no. 12242S, CST, USA), IL-6 (1:1000, catalog no. DF6087, Affinity Biosciences, China), stat-3 (1:1000, catalog no. 12640S, CST, USA), p-stat-3 (S727) (1:1000, catalog no. ET1607-39, Huabio, China), non-phospho (Active) β-catenin (1:1000, catalog no. 8814S, CST, USA) and TNF-α (1:1000, catalog no. 41504, Signalway Antibody, USA). After incubation with the primary antibodies overnight at 4°C, the signals were detected, and protein band intensities were analyzed via densitometry in Image Lab and quantified in ImageJ software separately.

### Enzyme-Linked Immunosorbent Assay (ELISA) Analysis

Cytokines including IL-1β, IL-6, TNF-α and IFN-γ in serum or intestinal tissue, and LPC in feces were measured by ELISA kits provided by FANKEL (Shanghai, China) according to manufacturer's instructions.

### Hematoxylin and Eosin (HE) Staining

The paraffine embedded specimens of colon were cut into 4-μm sections. After dewaxed, the sections were stained with hematoxylin and eosin for 3-5 min, and then photographed and analyzed under PANNORAMIC (3DHISTECH, Hungary) and CaseViewer2.4 (3DHISTECH, Hungary). As previously reported^14^, the histology score consisted of a composite scale which indicated the overall degree of inflammation and dysplasia.

### Immunohistochemistry Staining

Immunohistochemistry was performed as described previously^14^. Antibodies used were listed as below: PCNA antibody (catalog no. GB11010-1, Wuhan Servicebio Technology, China), β-catenin (abcam no. ab32572, Wuhan Servicebio Technology, China). Then, the paraffin sections were digitally scanned and analyzed under PANNORAMIC (3DHISTECH, Hungary) and Halo (Indica labs, USA).

### Immunofluorescence

For immunocytofluorescence, cells were fixed for 20 min in 4% paraformaldehyde (Biosharp, China) at room temperature for 20 min. After another two washing steps with PBS, the cells were blocked with blocking solution (Dawenbio, China) at room temperature for 1 h. Subsequently, the cells were washed twice with PBS. Samples were incubated at 4℃ overnight with primary antibodies (Occludin (1:200), ZO-1 (1:200) and E-cadherin (1:200, catalog no. ab40772, Abcam, USA)) and then at room temperature for 1h with secondary antibody (1:200, catalog no. ab150077, Abcam, USA). The nuclei were stained with 1μg/mL DAPI (Solarbio, China). Samples were scanned with confocal laser scanning microscopy (Leica TCS SP8). Five random fields from cell samples were collected for further analysis.

### Gut Microbiota Analysis

Bacterial genomic DNA was extracted from fecal samples with an E.Z.N.A.®Stool DNA Kit (Omega, USA) according to the manufacturer's instructions. Subsquently, 16S rDNA genes analysis was performed as previously described^14^. In brief, the V3 - V4 region of the 16S rRNA was amplified by PCR with the forward and reverse primers 341 (5'-CCTACGGGNGGCWGCAG-3') and 805R (5'-GACTACHVGGGTATCTAATCC-3'), respectively. After PCR amplification, purification was performed with an Agencourt AMPure XP purification kit (Beckman Coulter Inc., Brea, CA, USA), and quantification was performed with a Qubit (Invitrogen, USA) instrument. Amplicons were then verified with an Agilent 2100 Bioanalyzer (Agilent, USA) and Illumina sequencing (Kapa Biosciences, Woburn, MA, USA), and the final results were further analyzed with the NovaSeq PE250 platform.

### High-Throughput Transcriptome Sequencing (RNA-Seq) Detection

Total RNAs of colonic mucosa from HFD-fed mice and HFD+BBR treated mice were sent to LC-Bio Technology (Hangzhou, China) for sequencing and analysis. Briefly, RNA was extracted with Trizol reagent (Invitrogen, USA) according to the manufacturer's procedure. After the isolated RNA was purified by DNase, the quality and purity of RNA collected were checked through the bioanalyzer 2010 and RNA 1000 Nano LabChip Kit (Agilent, USA). Then, according to the protocol of RNA-Seq sample preparation kit (Illumina, San Diego, USA), the cleaved RNA was reverse transcribed to cDNA, and paired terminal sequencing was performed on the illumina Nova seq6000 (LC Sciences, USA), as described previously^14^.

### Metabolomic Profiling

The collected intestinal tissue samples for metabonomic analysis. The metabolites were extracted with 120 μL of precooled 50% methanol, vortexed mixed for 1 min, and incubated at room temperature for 10 min. After centrifugation at 4,000 g for 20 min, the supernatants were transferred into new 96 well plates. The samples were stored at -80 °C before the LC-MS analysis. First, all chromatographic separations were performed with an ultra-performance liquid chromatography (UPLC) system (SCIEX, UK). An ACQUITY UPLC T3 column (100 mm * 2.1 mm, 1.8 µm, Waters, UK) was used for the reversed phase separation. A high-resolution TripleTOF 5600plus tandem mass spectrometer (SCIEX, UK) was used to detect metabolites eluted from the column. The Q-TOF was operated in both positive and negative ion modes. LC-MS raw data files were converted into mzXML format and then processed with the XCMS, CAMERA, and metaX toolbox implemented in the R software.

### Cell Culture

Lysophosphatidylcholine (LPC) was purchased from Avanti Polar Lipids (Merch, Darmastadt, Germany). The normal intestinal epithelial cell line IEC-6 cell and the CRC cell lines (HCT-116, HCT-8 and SW480 cells) were seeded at 20,000 cells/well in a 24-well plate. Cells were serum starved for 24 hours and grown in RPMI 1640 medium supplemented with either 0.1% fatty acid-free bovine serum albumin or LPC (10 μmol/L and 20 μmol/L) in 0.1% fatty acid-free bovine serum albumin for up to 3 days. For cell counting, cells were trypsinized, and the number of cells was recorded at the end of the experiment. Cells were fixed in 70% ethanol overnight, stained with propidium iodide, and analyzed with flow cytometry. For western blotting analysis, 50,000 cells were seeded per well of a 12-well plate and treated as indicated. Finally, cells were rinsed, scraped, and collected for western blotting analysis.

RAW 264.7 mouse macrophages were obtained from the Chinese Academy of Sciences Cell Bank and cultured at 37 °C under 5% CO_2_/95% air in Dulbecco's Modified of Eagle's Medium (DMEM) containing 10% (v/v) heat inactivated FBS. A total of 5×10^5^ cells were seeded per well of a 24-well plate with (10 μmol/L and 20 μmol/L) or without LPC, and grown for up to 3 days. Subsequently, cells were collected for western blotting analysis.

To establish a cell co-culture system, we used Transwell chambers (0.4 μm, 6.5mm). The bottom chamber was filled with HCT-116, HCT-8 or SW480 cells, and RAW 264.7 mouse macrophages were suspended in the upper chamber. After culturing for 3 days in a 5% CO_2_, 37 °C incubator, cells in the bottom chamber were collected for immunofluorescence.

### Statistical Analysis

SPSS 23.0 software (IBM, Chicago, IL, USA) was used to analyze the data, and the normality and homogeneity of variance of the data were tested before further analysis. Generally, for comparisons of more than two groups, an analysis of variance (ANOVA) was used. Differences between two groups were performed with t-test analysis. When the data were skewed or the variance was not uniform, nonparametric tests were used to analyze significant differences between groups. Comparisons of categorical variables between 2 groups were performed using chi-square test or Fisher exact test. Graphing was performed in GraphPad Prism 8.0 software (La Jolla, CA, USA). All the data were shown as the mean ± SD.* P*-value less than 0.05 was considered statistically significant.

## Results

### BBR inhibits HFD-associated colorectal tumourigenesis in mice

To study the effect of BBR on HFD-associated colorectal tumourigenesis, we fed AOM/DSS treated Balb/c mice an HFD, with or without BBR administration (Figure 1A). The survival rate was significantly higher in BBR treated HFD-fed mice than in control mice receiving only the HFD (*p* < 0.05, Figure 1B). The colon length was longer (*p* < 0.05), and the colorectal tumor number was lower (*p* < 0.001), in HFD-fed mice with than without BBR (Figure 1C-E). On the basis of microscopic histological examination, the histology scores in mice fed an HFD were significantly higher than those in mice fed an ND or HFD+BBR (both *p* < 0.01, Figure 1F). The proliferation of colonic epithelial cells in HFD-fed mice was greater than in the ND-fed mice (*p* < 0.01) and HFD+BBR-fed mice (*p* < 0.001), as manifested by markedly higher histochemistry scores for proliferating cell nuclear antigen (PCNA), a marker of proliferating cells (Figure 1G).

### BBR suppresses inflammatory cytokines and repairs gut barrier function in AOM/DSS-treated mice fed an HFD

**Figure 2:**
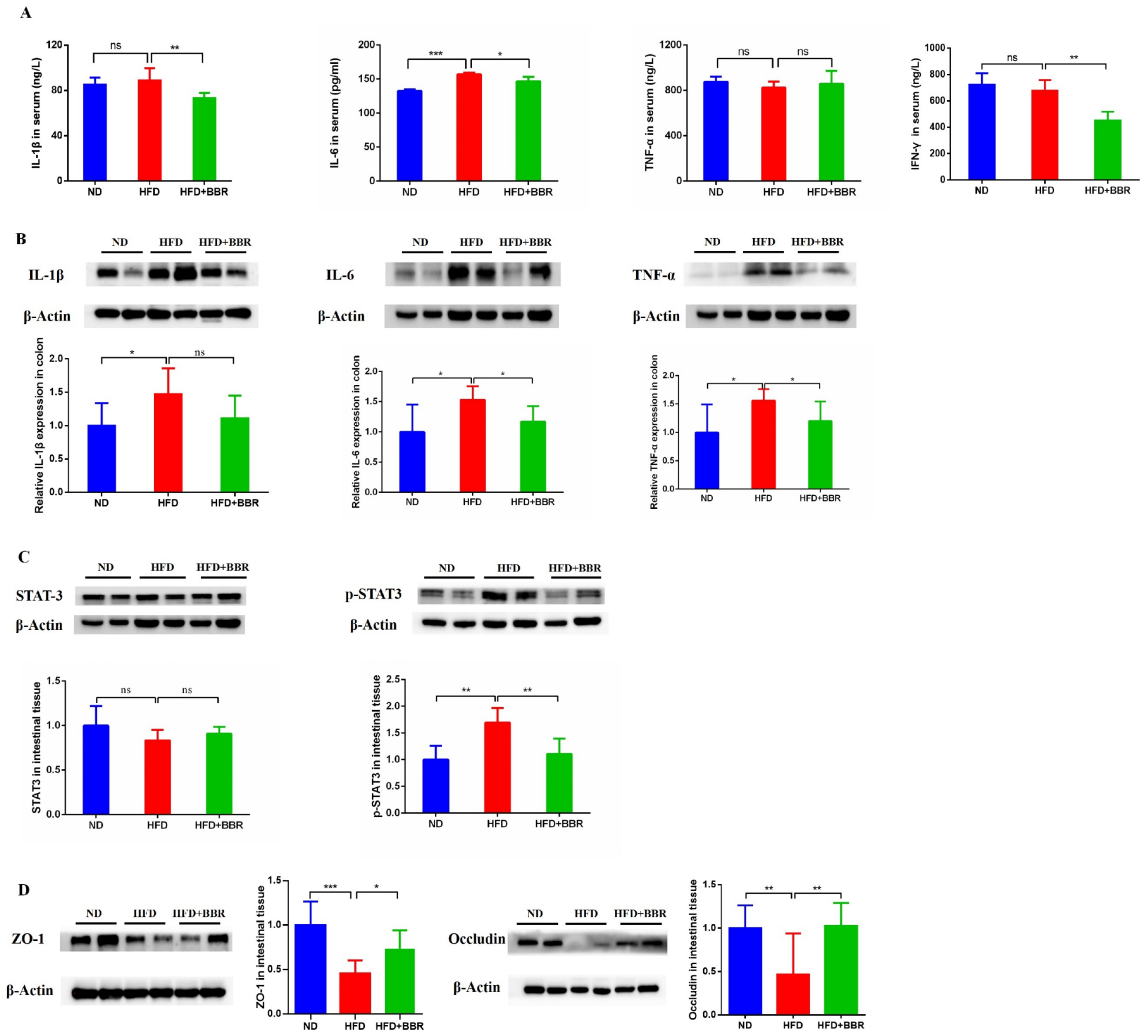
BBR suppresses inflammatory cytokines and repairs gut barrier function in CRC model mice fed with HFD. (A) Levels of inflammatory cytokines (IL-1β, IL-6, TNF-α and IFN-γ) in the serum. (B) Levels of inflammatory cytokines (IL-1β, IL-6, and TNF-α) in the colon tissues. (C) Expression of STAT3 and p-STAT3 in the colon tissues. (D) Expression of the gut barrier-associated proteins ZO-1 and occludin in colon tissues. (*p < 0.05, ^**^p < 0.001, ^***^p < 0.001).

To investigate the effects of BBR on inflammatory response, we measured the levels of inflammatory cytokines in the serum and colon. The level of serum interleukin-6 (IL-6) was markedly higher in HFD-fed mice than ND-fed mice (*p* < 0.001). However, the serum IL-1β (*p* < 0.01), IL-6 (*p* < 0.05), and INF-γ (*p* < 0.01) concentrations were significantly decreased after BBR treatment in HFD-fed mice (Figure 2A). Additionally, IL-1β, IL-6, and tumor necrosis factor-α (TNF-α) in colonic tissue were more elevated in HFD-fed mice than ND-fed mice (all *p* < 0.05), and BBR clearly suppressed the expression of colon IL-6 and TNF-α in HFD-fed mice, according to western blotting (all *p* < 0.05, Figure 2B). Previous studies have reported that activation of the IL-6/STAT3 signaling pathway was involved in the development of colitis and colitis-associated cancer^15^, ^16^. Thus, we examined the expression of STAT3 and phospho-STAT3 (p-STAT3) in the colonic epithelium. Clear p-STAT3 elevation was observed in HFD-fed mice compared with ND-fed mice, and BBR effectively inhibited p-STAT3 expression in colonic tissue (*p* < 0.01, Figure 2C).

Moreover, studies have shown that an HFD impaired gut barrier function^6^, ^17^. To confirm whether BBR might repair the gut barrier function damage induced by HFD in AOM/DSS treated mice, we observed the expression of colon tight junction proteins in HFD-fed mice with or without BBR treatment. The levels of Zonula occludens-1 (ZO-1) (*p* < 0.001) and occludin (*p* < 0.01) in colonic tissue were significantly lower in HFD-fed mice than ND-fed mice (Figure 2D). After BBR treatment of HFD-fed mice, the expression of ZO-1 (*p* < 0.05) and occludin (*p* < 0.001) were markedly increased, on the basis of western blotting (Figure 2D). These results indicated that BBR may repair gut barrier function via suppressing inflammatory pathways, such as the IL-6/p-STAT3 signaling pathway, thereby inhibiting HFD-associated colorectal tumourigenesis.

### BBR regulates gut microbiota dysbiosis induced by an HFD in AOM/DSS treated mice

**Figure 3:**
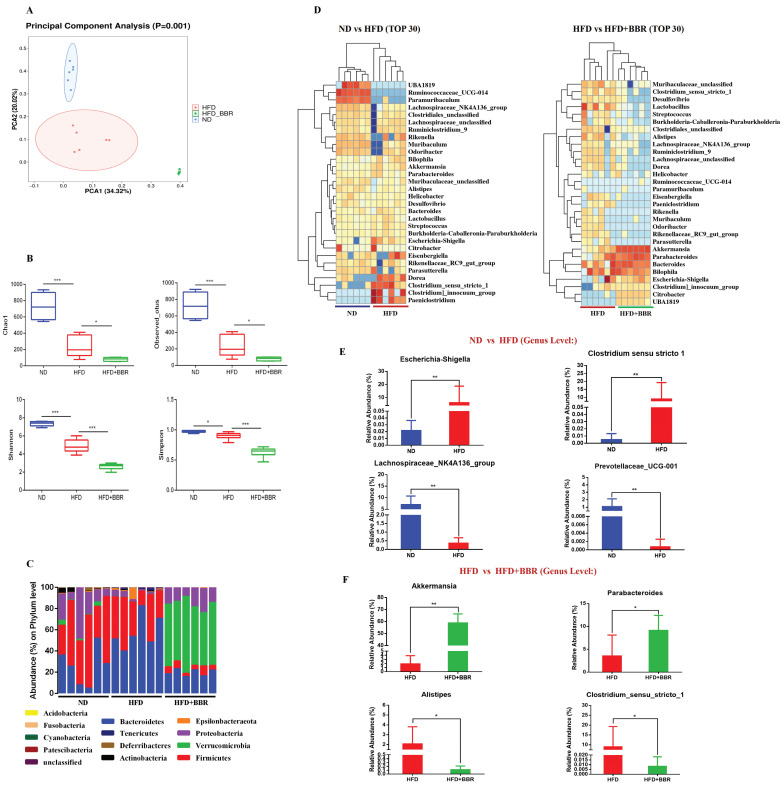
BBR regulates gut microbiota dysbiosis induced by HFD in AOM/DSS treated mice. (A) Principal component ordination analysis of the gut microbiota among the three groups. (B) Richness indices (Chao1 and Observed_outs) and diversity indices (Shannon and Simpson) of gut microbiota among three groups. (C) Bacterial taxonomic profiling at the phylum level among samples. (D) Relative abundance heat maps of the top 30 enriched bacterial OTUs between ND mice and HFD mice, or HFD mice and HFD+BBR mice. (E) Relative abundance of *Escherichia-Shigella*, *Clostridium_sensu_stricto_1*, *Prevotellaceae_UCG-001*, and *Lachnospiraceae_NK4A136_group* in stool samples of ND mice and HFD mice. (F) Relative abundance of *Akkermansia*, *Parabacteroides*, *Alistipes*, and *Clostridium_sensu_stricto_1* in stool samples of HFD mice and HFD+BBR mice. (*p < 0.05, ^**^p < 0.001).

To explore the potential role of gut microbiota alterations in BBR's suppression of HFD-associated CRC development, we performed 16S rRNA sequencing on fecal samples from ND-fed, HFD-fed, and HFD+BBR-fed AOM/DSS treated mice. PCA analysis (beta diversity) indicated significant differences in the clustering of the gut microbiota among the three groups (*p* = 0.001, Figure 3A). Lower bacterial richness (*p* < 0.001) and bacterial diversity (*p* < 0.05) were observed in HFD-fed mice than ND-fed mice (Figure 3B). Interestingly, BBR treatment further significantly decreased the bacterial richness (*p* < 0.05) and diversity (*p* < 0.001) in AOM/DSS mice fed an HFD (Figure 3B). Specifically, the abundance of *Verrucomicrobia* at the phylum level was enriched obviously after BBR treatment in mice fed with HFD (Figure 3C). The abundance of the top 30 genera with total coverage exceeding 85% (between ND-fed and HFD-fed mice) or 93% (between HFD-fed and HFD+BBR-fed mice) was visualized with a heat map (Figure 3D). The abundances of potential pathogenic bacterial genera including *Escherichia-Shigella* and *Clostridium_sensu_stricto_1* was significantly enriched, and that of beneficial bacteria such as *Prevotellaceae_UCG-001* and *Lachnospiraceae_NK4A136_group* was lower in HFD-fed mice than ND-fed mice (both *p* < 0.01); Interestingly, the protective bacterial genera, *Akkermansia* (*p* < 0.01) and *Parabacteroides* were enriched, and potentially pathogenic bacterial including *Alistipes* and *Clostridium_sensu_stricto_1* markedly decreased after BBR administration in mice fed with HFD (others *p* < 0.05, Figure 3F, Table S1). Together, these results suggested that the gut microbiota may play an important role in mediating BBR's effect against HFD-associated CRC development.

### BBR alters gut microbiota related metabolites in AOM/DSS treated mice fed with HFD

**Figure 4:**
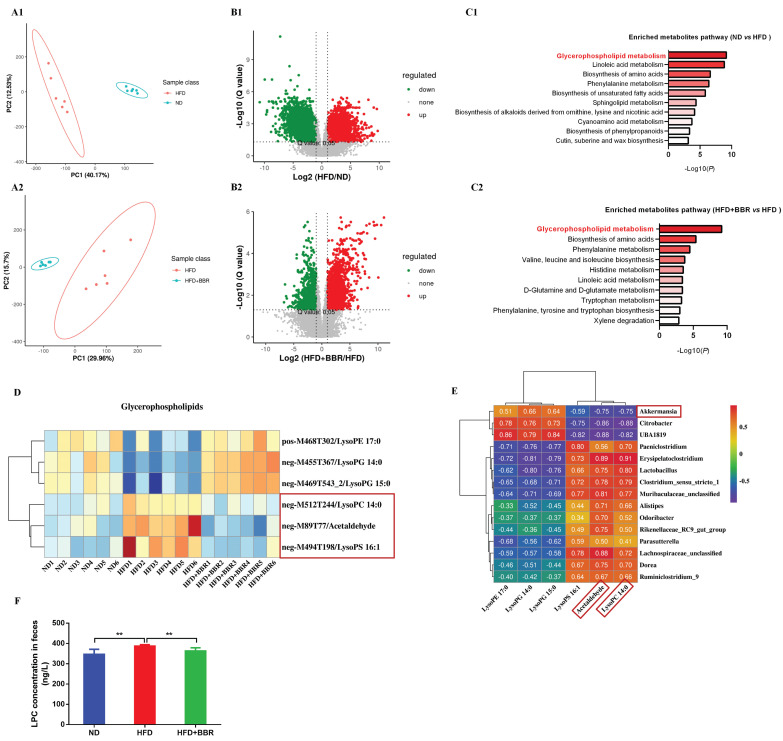
BBR alters gut microbiota related metabolites in AOM/DSS treated mice fed with HFD. (A1-2) The PCA plot of gut metabolomics analysis in ND mice and HFD mice, or HFD mice and HFD+BBR mice. (B1-2) Volcano plot of differential metabolites between ND mice and HFD mice, or HFD mice and HFD+BBR mice. (C1-2) Pathway analysis of differentially enriched metabolites in ND mice and HFD mice, or HFD mice and HFD+BBR mice. (D) Differential metabolites heat map for the glycerophospholipid metabolism pathway among the three groups. (E) Correlation analysis of the associations between the BBR-altered microorganisms and metabolites. (F) Mouse stool LPC concentrations in the three groups. (^**^p < 0.01).

Considering the regulatory roles of the microbiota in metabolites, we performed metabolic profiling of the fecal samples from ND-fed, HFD-fed, and HFD+BBR-fed mice treated with AOM/DSS by using LC-MS, to uncover metabolic changes induced by BBR treatment in HFD-associated CRC model mice. The fecal metabolic profiles significantly differed between ND-fed and HFD-fed mice (Figure 4A1), and between HFD-fed and HFD+BBR-fed mice (Figure 4A2), according to unsupervised PCA analysis. Differential fecal metabolites were also identified between two groups (Figure 4B1-2), and were found to be enriched in different metabolomic signaling pathways. Among these pathways, glycerophospholipid metabolism was the top altered pathway in mice fed with HFD compared to that fed with ND mice (Figure 4C1). Interestingly, glycerophospholipid metabolism was also the top altered pathway in mice fed with HFD after BBR treatment (Figure 4C1). Through further analysis of the top pathway, lysophosphatidylcholine (LPC), acetaldehyde and lysophosphatidic acid (LPA) were found to significantly decrease in HFD-fed mice after BBR administration (Figure 4D, Table S2).

To determine the potential association between microbiota and metabolites, we analyzed the relationships among differentially present bacteria and metabolites through partial Spearman's correlation analysis. *Akkermansia* had the highest abundance among bacterial genera after BBR treatment and had the most negative correlation with LPC and acetaldehyde, whereas two pathogenic bacteria, *Clostridium_sensu_stricto_1* and *Alistipes*, were enriched in HFD-fed mice, and were positively associated with LPC and acetaldehyde (Figure 4E). LPC has been documented to exert epigenetic effects often associated with tumourigenesis^18^, ^19^. Therefore, we verified that the LPC content in the intestine of mice fed with HFD was significantly higher than that in mice fed with ND, and that BBR treatment clearly suppressed the content of LPC in HFD-fed mice (both *p* < 0.01, Figure 4F).

### The BBR-associated differential metabolite LPC contributes to cell proliferation and cell junction impairment

**Figure 5:**
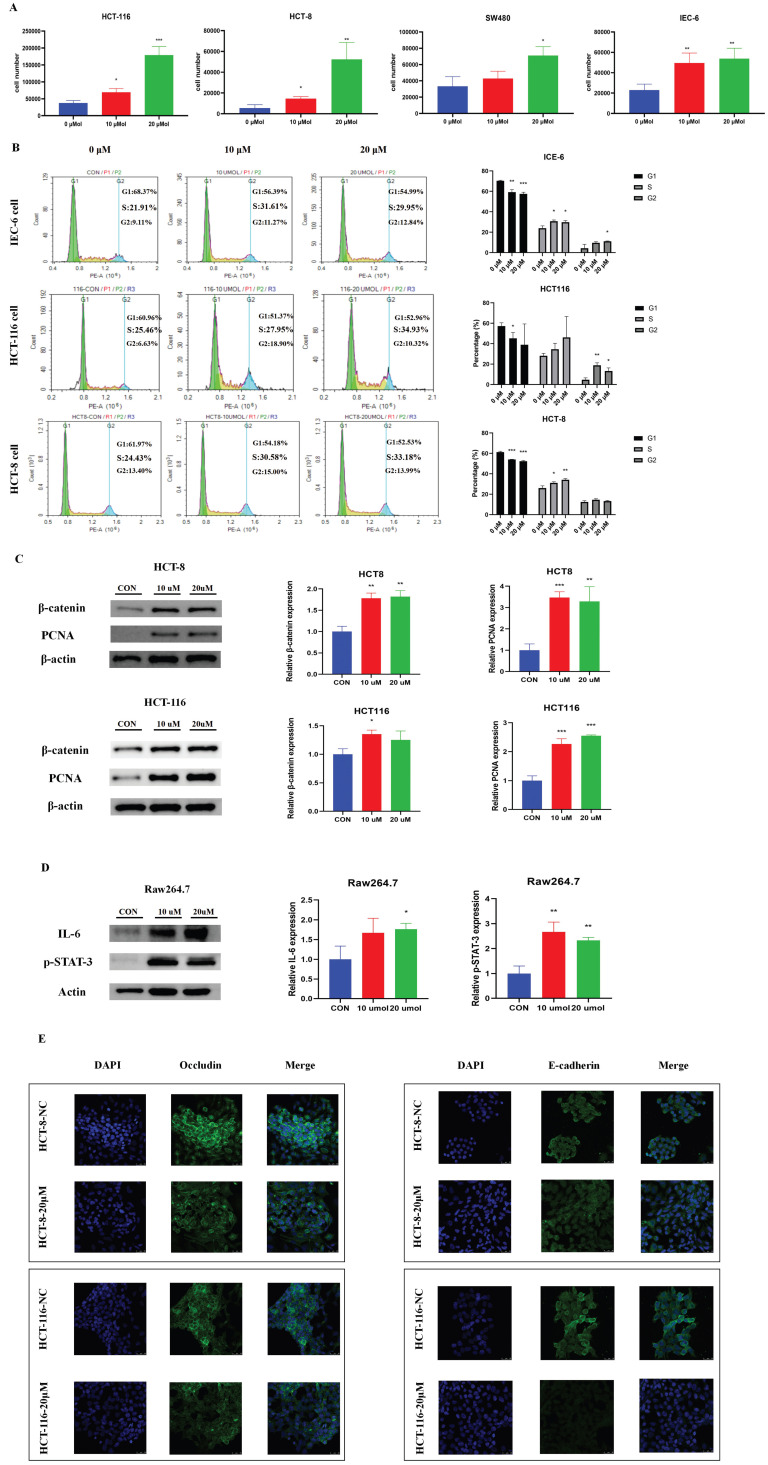
The BBR-associated differential metabolite LPC contributes to cell proliferation and cell junction impairment. (A) Cell growth curves of the CRC cell lines HCT116, HCT-8, SW480, and the normal intestinal epithelial cell line IEC-6, with or without LPC treatment. (B) HCT 116 cells and HCT 8 cells with or without LPC treatment were analyzed with flow cytometry. (C) Expression of β-catenin and PCNA in HCT 8 cells and HCT 116 cells with or without LPC treatment. (D) Expression of IL-6 and p-STAT3 in Raw 264.7 cells with or without LPC treatment. (E) Expression of occludin and E-cadherin in HCT-8 cells and HCT-116 cells co-cultured with Raw264.7 cells with or without LPC treatment. (^*^p < 0.05, ^**^p < 0.001, ^***^p < 0.001).

To explore the potential functional roles of LPC in CRC development, we directly treated three CRC cell lines (HCT-116, HCT-8 and SW480) and a normal intestinal epithelial cell line (IEC-6) were directly treated with LPC. LPC obviously promoted proliferation of CRC cells and normal intestinal epithelial cell (Figure 5A). Cell cycle experiments suggested that LPC treatment, compared with a negative control, accelerated the cell cycle progression of IEC-6, HCT 116, and HCT-8 cells from G1 to S phase (Figure 5B). In addition, the expression of β-catenin and PCNA significantly increased after LPC administration in HCT-116 and HCT-8 cells (Figure 5C, Figure S1).

Moreover, to confirm whether LPC has a regulatory role on inflammatory pathways and intestinal epithelial barrier function, we used LPC to treat Raw264.7 cells. IL-6 and p-STAT3 proteins were obviously up-regulated by LPC treatment (Figure 5D), thus indicating that LPC plays a catalytic role in the IL-6/p-STAT3 pathway. Subsequently, we co-cultured Raw264.7 cells with HCT-8 or HCT-116 cells. Immunofluorescence indicated that LPC treatment markedly decreased the proteins of occludin and E-cadherin in HCT-8 or HCT-116 cells (Figure 5E). In summary, these results indicated that the inhibitory role of BBR in the progression of HFD-associated CRC, occurred at least partly through the regulation of glycerophospholipid metabolism through suppressing the content of LPC in the intestines.

### BBR inhibits oncogenic pathways and pro-inflammatory signaling genes in HFD-fed mice

**Figure 6:**
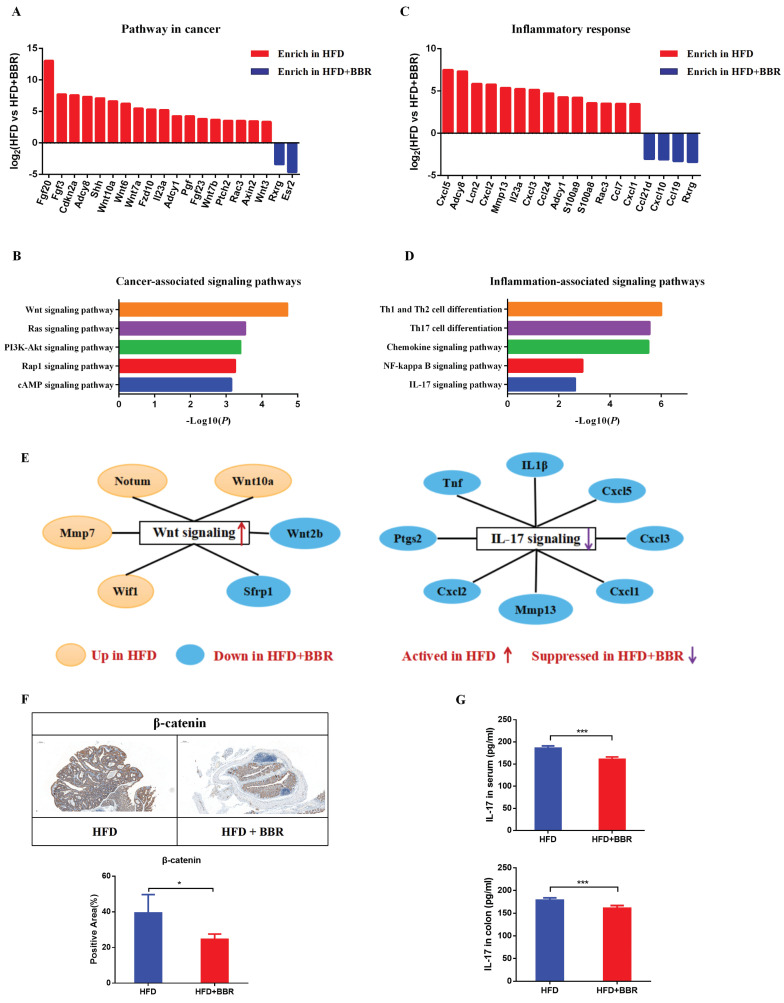
BBR inhibits oncogenic pathways and pro-inflammatory signaling genes in HFD-fed mice. (A) Differentially expressed genes of the colonic epithelium involved in cancer pathways in the HFD group compared with the HFD+BBR group. (B) Altered cancer signaling pathways in the HFD group compared with the HFD+BBR group, according to enrichment analysis. (C) Differentially expressed genes of the colonic epithelium involved in inflammatory response pathways in the HFD group compared with the HFD+BBR group. (D) Altered inflammatory response signaling pathways in the HFD group compared with HFD+BBR group, according to enrichment analysis. (E) The differentially expressed genes in the Wnt signaling pathway and Th 17 signaling pathway, shown in a network. (F) Expression of β-catenin in colon tissues: bar=200μm, (40μm × 5). (G) Levels of IL-17 in the serum and colon tissues between groups. (^*^p < 0.05,^ ***^p < 0.001).

To comprehensively understand the molecular mechanism through which BBR inhibits HFD-associated colorectal tumourigenesis, we performed RNA sequencing analysis of the gene expression profiles of colonic epithelium in HFD-fed mice with or without BBR treatment. The differentially expressed genes involved in pathways in cancer and pro-inflammatory responses were identified with |logFC| >3 and adjusted *p* < 0.01. The data for cancer associated genes revealed 18 up-regulated and 2 down-regulated genes in HFD-fed mice compared to HFD-fed mice treated with BBR (Figure 6A, Table S3). Enrichment analysis indicated that Wnt/β-catenin signaling was the main pathway through which BBR inhibits intestinal tumorgenesis (Figure 6B). In addition, the data for pro-inflammatory response genes indicated 14 up-regulated and 4 down-regulated genes in HFD-fed mice compared with HFD-fed mice with BBR treatment (Figure 6C, Table S4). Meanwhile, multiple pathways, including NF-kappa B signaling and IL-17 signaling, were found to be involved in BBR's anti HFD-associated colorectal tumourigenesis (Figure 6D). Furthermore, significantly lower protein expression of β-catenin and IL-17 was observed in HFD-fed mice treated with BBR than in HFD-fed mice, according to immunohistochemistry and ELISA analysis, respectively (Figure 6E-G). Together, the results suggested that BBR inhibits HFD-associated colorectal cancer through suppressing the Wnt and IL-17 signaling pathways.

### Antibiotics attenuate the inhibitory effect of BBR on HFD-associated colorectal tumourigenesis in mice

**Figure 7:**
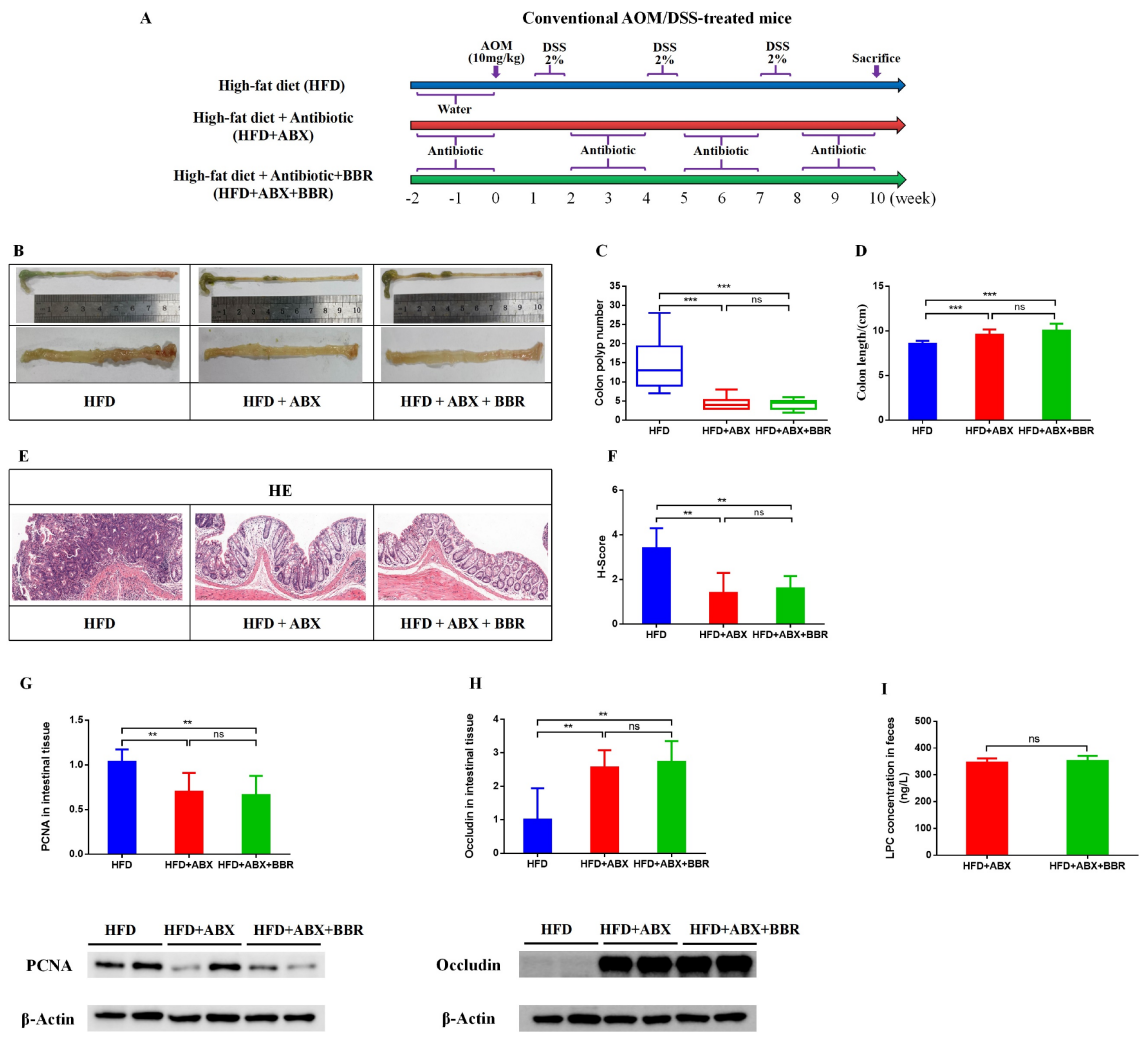
Antibiotic treatment attenuates the inhibitory effect of BBR on HFD-associated colorectal tumourigenesis in mice. (A) Design of antibiotic experiment on AOM/DSS mice fed with HFD. (B) Representative images of the colon at sacrifice. (C) Number of colon polyps. (D) Colon length. (E) HE staining of intestinal sections: bar=50μm, (10μm × 5). (F) Histology scores among the three groups. (G) Expression of PCNA in colon tissues. (H) Expression of occludin in colon tissues. (I) Mouse stool LPC concentrations among the three groups. (^**^p < 0.001, ^***^p < 0.001)

To determine whether the gut microbiota is involved in BBR's inhibition of HFD related colorectal tumourigenesis, we used antibiotics to deplete the gut microbiota, and then observed the regulatory effects of BBR on the progression of intestinal tumors in HFD-fed mice (Figure 7A). As in previous studies^6^, ^20^, antibiotics effectively decreased the number of colon polyps and alleviated the shortening of the colon length in HFD-fed mice. However, no differences were observed in colon length and colon polyp numbers between HFD+ABX mice and HFD+ABX+BBR mice (Figure 7B-D). HE staining showed that antibiotics markedly decreased the histology scores of the intestinal mucosa in HFD-fed mice (*p* < 0.01), whereas BBR did not further decreased the histological scores after antibiotics treatment (Figure 7E, F). Meanwhile, in colonic tissue, the protein expression of PCNA was significantly lower, and that of occludin was markedly higher in HFD+ABX mice and HFD+ABX+BBR mice than in HFD group mice (*p* < 0.01), whereas no difference was observed in antibiotics treated HFD-fed mice with or without BBR administration (Figure 7G, H). Additionally, to clarify the effect of antibiotics on BBR's regulation of LPC expression, we determined the content of LPC in the intestines with or without BBR treatment in HFD-fed mice under antibiotics intervention, and observed no significant differences (Figure 7I). These results indicated that the gut microbiota mediates the inhibitory effect of BBR on HFD-associated colorectal tumourigenesis.

### FMT recapitulate the inhibitory effects of BBR on colorectal tumourigenesis in mice fed with HFD

**Figure 8:**
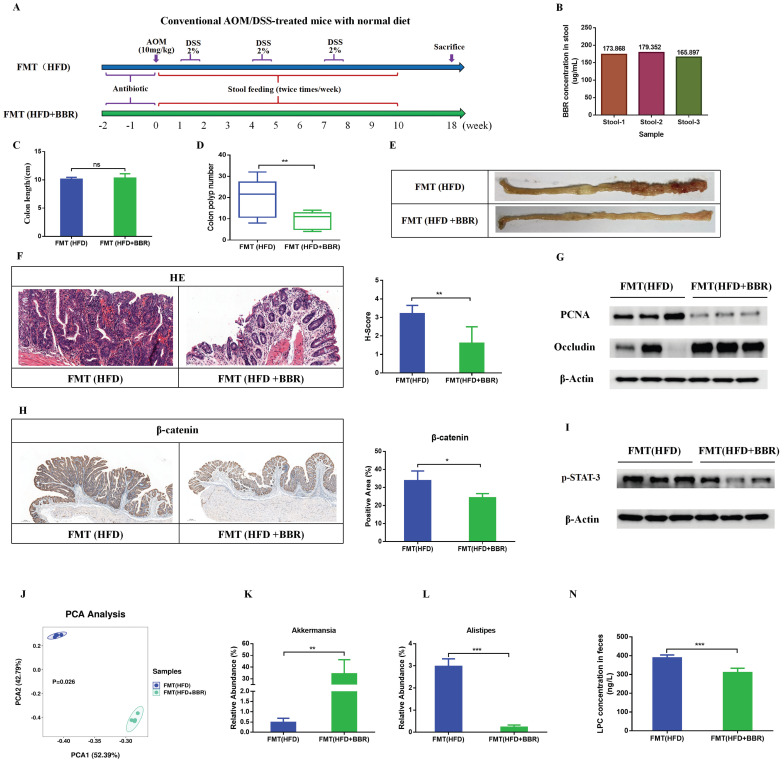
FMT recapitulates the inhibitory effects of BBR on colorectal tumourigenesis in mice fed with HFD. (A) Design of FMT experiment on AOM/DSS mice. (B) BBR concentration in the stool in two groups of mice. (C) Colon length. (D) Number of colon polyps. (E) Representative images of the colon at sacrifice. (F) HE staining of intestinal sections and histology scores: bar=50μm, (10μm × 5). (G) Expression of PCNA and occludin in colon tissues. (H) IHC staining of colorectal sections, showing the expression of β-catenin: bar=200μm, (40μm × 5). (I) Expression of p-STAT3 in colon tissues. (J) Principal component ordination analysis of the gut microbiota between the two groups. (K) Relative abundance of *Akkermansia* in stool samples of FMT(HFD) mice and FMT(HFD+BBR) mice. (L) Relative abundance of *Alistipes* in stool samples of FMT(HFD) mice and FMT(HFD+BBR) mice. (N) Mouse stool LPC concentrations in the two groups. (^*^p < 0.05, ^**^p < 0.001, ^***^p < 0.001).

To further validate the contribution of the BBR regulated gut microbiota to HFD-associated colorectal tumourigenesis, we transferred fecal samples from HFD-fed mice, with or without BBR administration, to AOM/DSS-induced CRC mice with microbiota depletion through antibiotics treatment (Figure 8A). Given that BBR bioavailability is very low in vivo^21^, ^22^, high concentrations of BBR might have remained in the stool. To determine the content of BBR remaining in the stool before FMT, we used LC-MS to confirmed that the content of BBR remaining in fecal suspension was approximately 170 ug/ml on the basis of comparison with a BBR standard sample, which was significantly lower than the concentration of BBR (100mg/kg/d) directly treated in HFD-fed mice (Figure 8B). Therefore, the FMT experiments excluded a direct anti-tumor effect of BBR treatment. Although FMT(HFD) mice showed no difference in colon length with respect to FMT(HFD+BBR) mice (Figure 8C), stools from the HFD+BBR group mice led to markedly decreased colonic polyp numbers (*p* < 0.01) and lower intestinal mucosa histological scores (*p* < 0.01) in FMT(HFD+BBR) mice than FMT(HFD) mice (Figure 8D-F). Moreover, stools from HFD+BBR mice, compared with HFD-fed mice, significantly suppressed the PCNA expression and increased occludin in colonic tissue (Figure 8G). Mechanically, to understand how BBR regulated gut microbiota/metabolites inhibited HFD-associated colonic proliferation, we further examined the oncogenic and inflammatory pathways closely associated with the effects of BBR against HFD-associated colorectal tumourigenesis. The expression of β-catenin and p-STAT3, which are involved in the Wnt and IL-6/STAT3 signaling pathways, was lower in FMT(HFD+BBR) mice than FMT(HFD) mice (Figure 8H, I). These data indicated that BBR-regulated gut microbiota/metabolites directly inhibited HFD-associated colorectal tumourigenesis and repaired gut barrier damage.

To verify whether the microbiota successfully colonized in the gut after FMT, we used 16S rRNA sequencing to determine the structure and composition of the gut microbiota after FMT treatment. The composition of the gut microbiota significantly differed between FMT(HFD) mice and FMT(HFD+BBR) mice (Figure 8J). Furthermore, we evaluated the abundance of genus level and found enrichment in the beneficial bacteria *Akkermansia* in FMT(HFD+BBR) mice (*p* < 0.01, Figure 8K), and in the potential pathogenic *Alistipes* in FMT(HFD) mice (*p* < 0.001, Figure 8L). Moreover, to clarify the regulatory effects of the gut microbiota on metabolites, we detected the LPC levels in recipient mice and found that stools from HFD+BBR mice resulted in significantly lower LPC content than stools from HFD-fed mice (*p* < 0.001, Figure 8N). These findings, combined with the microbiota results, clarified the roles of both the altered gut microbiota and its related metabolite LPC in BBR's effects against HFD-associated colorectal tumourigenesis.

## Discussion

Clinical studies have confirmed that a Western diet is associated with an elevated risk of CRC in humans. Epidemiological data have indicated that an HFD often leads to obesity and increases the risk of colon cancer in men by 30-70%^23^. Meanwhile, growing evidence indicates that HFD promotes colorectal tumourigenesis by increasing intestinal inflammation through modulating the gut microbiota and related metabolites^6^, ^24^. Therefore, effective drugs are urgently needed for the prevention and treatment of HFD-associated CRC. BBR, a regulator of the gut microbiota, has been reported to increase the abundance of beneficial bacteria while suppressing the abundance of pathogenic bacteria^14^, ^25^. BBR is currently widely used in the treatment of intestinal infection, and has been found to be safe and effective in decreasing the risk of colorectal adenoma recurrence in clinical settings^26^. Although BBR has been observed to have substantial therapeutic potential for HFD-associated colorectal tumourigenesis, the therapeutic mechanism remains to be fully clarified. Notably, whether BBR altered the gut microbiota and associated metabolites was unclear.

Our study revealed that BBR effectively inhibited colorectal tumourigenesis by modifying the gut microbiota and its metabolites in HFD-fed mouse models. BBR was found to significantly inhibit intestinal inflammatory and oncogenic pathways, alleviate epithelial barrier damage, and enrich bacteria involved in glycerophospholipid metabolism in HFD-fed CRC model mice However, BBR treatment of mice with gut microbiota-depletion showed no significant effects against HFD-associated colorectal tumourigenesis. Notably, transplantation of feces from BBR-treated HFD-fed mice to CRC model mice with microbiota-depletion inhibited colorectal tumourigenesis and LPC concentration. To our knowledge, this study is the first to reveal the association between BBR and the gut microbiota in the regulation of glycerophospholipid metabolism and HFD-associated colorectal tumourigenesis.

BBR treatment significantly altered the structure of the gut microbiome in HFD-fed mice, mainly by changing the number of OTUs, microbiome diversity, and taxa richness. Specifically, BBR clearly enriched the abundance of *Verrucomicrobia* at the phylum level. Significant changes were also observed at the genus level; the relative abundances of *Escherichia-Shigella* and *Clostridium_sensu_stricto_1* enriched in HFD-fed mice, but, together with* Alistipes*, decreased in BBR treated HFD-fed mice, whereas beneficial gut microorganisms, such as *Akkermansia* and* Parabacteroides*, significantly increased.

The *Akkermansia*, genus had the highest abundance (over 55%) after BBR treatment in this study, and has been proved to suppress the inflammatory response, elevate SCFAs, and regulate immune functions in several studies^27^-^29^. Importantly, a growing body of evidence indicates that *Akkermansia* inhibits the occurrence and development of CRC^30^, ^31^. A recent study has reported that Baitouweng decoction significantly alleviates the inflammatory symptoms of mice with acute colitis by increasing *Akkermansia* and suppressing the IL-6/STAT3 pathway^32^. Similarly, our study showed that BBR effectively increased *Akkermansia* abundance and inhibited the L-6/STAT3 pathway in AOM/DSS mice fed with HFD, thus indicating a potential mechanism underlying BBR's prevention of tumor progression.

In addition, the abundance of *Alistipes, Escherichia-Shigella*, and* Clostridium_sensu_stricto_1* is greatly increased in patients with CRC, and colonization of these bacteria in CRC model mice has been found to accelerate tumor progression^33^-^35^. Multi-cohort analysis of the CRC metagenome has identified that *Alistipes* may be used as a potential bacterial marker for clinical CRC diagnosis^36^. Studies have confirmed that *Alistipes finegoldii*, a species of the *Alistipes*, promotes right sided CRC through activation of the IL-6/STAT3 pathway^37^. Moreover, an experimental study has found that high abundance of *Escherichia/Shigella* was closely associated with the activation of the Wnt pathway in Sirt3-deficient CRC model mice^38^. Given the strong association between *Clostridium_sensu_stricto_1* and inflammation-associated genes such as REG3G, CCL8, and IDO1, this bacterium appears to promote the development of colitis^39^. In our study, BBR treatment effectively decreased the abundance of the above pathogenic bacteria, and of intestinal inflammatory factors including IL-1β, IL-6, and INF-γ, and also suppressed the IL-6/STAT3 and Wnt signaling pathways in CRC model mice fed with HFD. Additionally, *Parabacteroides,* a promising probiotic candidate, has been demonstrated to have a protective role in colonic tumourigenesis and intestinal epithelial barrier maintenance in AOM-treated mice with HFD^40^.

Moreover, the *Akkermansia* genus, which had the highest enrichment after BBR treatment, was negatively associated with LPC. Although no definiteive evidence indicates that *Akermancia* directly regulates glycerophospholipid metabolism, relevant studies have reported that decreased relative abundance of *Akkermansia* is associated with increased content of LPC in ApoE^-/-^ mice with atherosclerosis co-depression^41^. Similarly, other studies have revealed that drugs regulate the abundance of *Akkermansia* while modulating the concentration of LPC in the gut^42^, ^43^. Together, these results indicate that *Akkermansia* may have a major regulatory effect on glycerophospholipid metabolism, particularly in regulating LPC concentration. Furthermore, a study of the effects of an HFD in promoting colorectal tumourigenesis has shown that *Parabacteroides* depletion is negatively associated with both LPC and LPA^44^. Therefore, our findings indicated that BBR against HFD-associated tumourigenesis was closely associated with alterations in the regulation of glycerophospholipid metabolism, in a manner dependent on the regulatory effect of BBR on the gut microbiota.

Recent studies have confirmed that glycerophospholipid metabolism was involved in the pathogenesis of various diseases, including obesity, hyperlipidemia, type 2 diabetes, colitis, and colorectal carcinogenesis^45^-^49^. Multiple studies have proved that glycerophospholipid metabolism was involved in the occurrence and development of CRC, and several drugs have anti-CRC therapeutic roles through regulating this metabolic pathway^50^, ^51^. More importantly, LPC, an important metabolite in glycerophospholipid metabolism, has been demonstrated to increase the inflammatory response in vitro and in vivo; in addition, its content in the intestines is regulated by the gut microbiota^52^. In our research, we discovered that the concentration of LPC was markedly elevated in HFD-fed mice with intact gut microbiota, and BBR effectively reversed this change. In addition, LPC effectively promoted CRC cell proliferation, hyperactivation of inflammatory cytokines, and impairment in epithelial cell junctions in vitro. Interestingly, in gut microbiome-depleted HFD-fed mice, BBR did not decrease the LPC concentration, and a loss of anti-tumor effects was observed. Taken together, these results indicated that BBR regulated HFD-induced gut microbiota and related metabolism dysregulation, thus decreasing the concentration of oncogenic LPC, suppressing cell proliferation, and improving the gut barrier, thereby inhibiting HFD-associated colorectal tumourigenesis.

Finally, we explored the direct effect of BBR regulated-gut microbiota on the colonic mucosa in gut microbiome-depleted mice fed an ND. Compared to mice receiving stool from HFD mice, mice receiving stool from HFD+BBR mice fed an ND had lower LPC concentrations in the gut, and showed significantly fewer colonic polyps and less cell proliferation, thus suggesting a direct role of BBR regulated-gut microbiota in contributing to the inhibition of CRC development. Mechanistically, we found that the BBR regulated-gut microbiota inhibited the IL-6/STAT3 and Wnt signaling pathways, which are involved primarily in regulating cell proliferation. Simultaneously, the BBR regulated gut microbiota also effectively improved epithelial cell junctions and protected intestinal integrity. Previous studies have shown that the gut microbiota regulates oncogenic genes and inflammatory factors^53^, ^54^. Futhermore, transplanting stools from BBR treated colitis mice to germ-free mice has been found to down-regulate intestinal inflammation and improve the intestinal mucosal barrier^25^, ^55^, thus supporting our observations in this study. Additionally, gut microbiota analysis indicated a significant difference in gut microbiota composition between HFD-FMT mice and HFD+BBR-FMT mice, thus indicating that regulation of the gut microbiota may be an important factor in BBR's improvement of intestinal barrier function and inhibition of tumor progression. Collectively, our fingdings indicated that oral BBR modulated the gut microbiota/metabolites imbalance caused by excessive dietary fat, thus inhibiting cell proliferation, alleviating gut barrier dysfunction, and ultimately preventing the progression of CRC development.

## Conclusion

Our study indicated that BBR treatment regulated the gut microbiota, suppressed LPC production, and inhibitd HFD-associated colorectal tumourigenesis. CRC model mice with gut microbiota depletion showed no difference in LPC concentration with or without BBR administration, and BBR treatment did not further inhibit HFD-associated tumor progression in antibiotic treated CRC model mice. Transplantation of fecal microbiota from HFD-fed mice and HFD-fed+BBR treated mice to gut microbiota depleted CRC model mice recapitulated the inhibitory effects of BBR on colorectal tumourigenesis and LPC levels, thereby indicating a direct link between BBR-regulated gut microbiota and HFD-associated colorectal tumourigenesis. These findings are the first to demonstrate that BBR inhibits HFD-associated CRC directly through modulating the gut microbiota-mediated LPC formation. Our data provided insights supporting the future application of BBR as a gut microbiota regulator in the clinical treatment of CRC, particularly for patients who prefer a Western diet.

## Supplementary Material

Supplementary figure and tables.

## References

[1] Siegel R L, Miller K D, Fuchs H E (2022). Cancer statistics, 2022. CA Cancer J Clin.

[2] Keum N, Giovannucci E (2019). Global burden of colorectal cancer: emerging trends, risk factors and prevention strategies. Nat Rev Gastroenterol Hepatol.

[3] Baena R, Salinas P (2015). Diet and colorectal cancer. Maturitas.

[4] Vernia F, Longo S, Stefanelli G (2021). Dietary Factors Modulating Colorectal Carcinogenesis. Nutrients.

[5] Sofi F, Dinu M, Pagliai G (2019). Fecal microbiome as determinant of the effect of diet on colorectal cancer risk: comparison of meat-based versus pesco-vegetarian diets (the MeaTIc study). Trials.

[6] Yang J, Wei H, Zhou Y (2022). High-Fat Diet Promotes Colorectal Tumorigenesis Through Modulating Gut Microbiota and Metabolites. Gastroenterology.

[7] Zhang W, Xu J H, Yu T (2019). Effects of berberine and metformin on intestinal inflammation and gut microbiome composition in db/db mice. Biomed Pharmacother.

[8] Zhang Y, Gu Y, Ren H (2020). Gut microbiome-related effects of berberine and probiotics on type 2 diabetes (the PREMOTE study). Nat Commun.

[9] Zhu L, Gu P, Shen H (2019). Protective effects of berberine hydrochloride on DSS-induced ulcerative colitis in rats. Int Immunopharmacol.

[10] Huang D N, Wu F F, Zhang A H (2021). Efficacy of berberine in treatment of rheumatoid arthritis: From multiple targets to therapeutic potential. Pharmacol Res.

[11] Xu X, Zhu X P, Bai J Y (2019). Berberine alleviates nonalcoholic fatty liver induced by a high-fat diet in mice by activating SIRT3. FASEB J.

[12] Chen H, Zhang F, Zhang J (2020). A Holistic View of Berberine Inhibiting Intestinal Carcinogenesis in Conventional Mice Based on Microbiome-Metabolomics Analysis. Front Immunol.

[13] Wang H, Guan L, Li J (2018). The Effects of Berberine on the Gut Microbiota in Apc (min/+) Mice Fed with a High Fat Diet. Molecules.

[14] Chen H, Ye C, Cai B (2022). Berberine inhibits intestinal carcinogenesis by suppressing intestinal pro-inflammatory genes and oncogenic factors through modulating gut microbiota. BMC Cancer.

[15] Xuan-Qing C, Xiang-Yu L V, Shi-Jia L (2021). Baitouweng decoction alleviates dextran sulfate sodium-induced ulcerative colitis by regulating intestinal microbiota and the IL-6/STAT3 signaling pathway. J Ethnopharmacol.

[16] Grivennikov S, Karin E, Terzic J (2009). IL-6 and Stat3 are required for survival of intestinal epithelial cells and development of colitis-associated cancer. Cancer Cell.

[17] Seguella L, Pesce M, Capuano R (2021). High-fat diet impairs duodenal barrier function and elicits glia-dependent changes along the gut-brain axis that are required for anxiogenic and depressive-like behaviors. J Neuroinflammation.

[18] Shimizu R, Kanno K, Sugiyama A (2015). Cholangiocyte senescence caused by lysophosphatidylcholine as a potential implication in carcinogenesis. J Hepatobiliary Pancreat Sci.

[19] Gao F, Chen J, Zhang T (2022). LPCAT1 functions as an oncogene in cervical cancer through mediating JAK2/STAT3 signaling. Exp Cell Res.

[20] Hwang S, Jo M, Hong J E (2020). Protective Effects of Zerumbone on Colonic Tumorigenesis in Enterotoxigenic Bacteroides fragilis (ETBF)-Colonized AOM/DSS BALB/c Mice. Int J Mol Sci.

[21] Younis F A, Saleh S R, El-Rahman S (2022). Preparation, physicochemical characterization, and bioactivity evaluation of berberine-entrapped albumin nanoparticles. Sci Rep.

[22] Zhou Y, Liu S Q, Peng H (2015). In vivo anti-apoptosis activity of novel berberine-loaded chitosan nanoparticles effectively ameliorates osteoarthritis. Int Immunopharmacol.

[23] Bardou M, Barkun A N, Martel M (2013). Obesity and colorectal cancer. Gut.

[24] Zeng H, Umar S, Rust B (2019). Secondary Bile Acids and Short Chain Fatty Acids in the Colon: A Focus on Colonic Microbiome, Cell Proliferation, Inflammation, and Cancer. Int J Mol Sci.

[25] Cui H, Cai Y, Wang L (2018). Berberine Regulates Treg/Th17 Balance to Treat Ulcerative Colitis Through Modulating the Gut Microbiota in the Colon. Front Pharmacol.

[26] Chen Y X, Gao Q Y, Zou T H (2020). Berberine versus placebo for the prevention of recurrence of colorectal adenoma: a multicentre, double-blinded, randomised controlled study. The lancet. Gastroenterology & hepatology.

[27] Gu Z, Pei W, Shen Y (2021). Akkermansia muciniphila and its outer protein Amuc_1100 regulates tryptophan metabolism in colitis. Food Funct.

[28] Yang W, Ren D, Zhao Y (2021). Fuzhuan Brick Tea Polysaccharide Improved Ulcerative Colitis in Association with Gut Microbiota-Derived Tryptophan Metabolism. J Agric Food Chem.

[29] Zhang T, Li Q, Cheng L (2019). Akkermansia muciniphila is a promising probiotic. Microb Biotechnol.

[30] Gu Z Y, Pei W L, Zhang Y (2021). Akkermansia muciniphila in inflammatory bowel disease and colorectal cancer. Chin Med J (Engl).

[31] Wang L, Tang L, Feng Y (2020). A purified membrane protein from Akkermansia muciniphila or the pasteurised bacterium blunts colitis associated tumourigenesis by modulation of CD8(+) T cells in mice. Gut.

[32] Xuan-Qing C, Xiang-Yu L V, Shi-Jia L (2021). Baitouweng decoction alleviates dextran sulfate sodium-induced ulcerative colitis by regulating intestinal microbiota and the IL-6/STAT3 signaling pathway. J Ethnopharmacol.

[33] Sheng Q, Du H, Cheng X (2019). Characteristics of fecal gut microbiota in patients with colorectal cancer at different stages and different sites. Oncol Lett.

[34] Wang T, Cai G, Qiu Y (2012). Structural segregation of gut microbiota between colorectal cancer patients and healthy volunteers. ISME J.

[35] Zeng X, Jia H, Zhang X (2021). Supplementation of kefir ameliorates azoxymethane/dextran sulfate sodium induced colorectal cancer by modulating the gut microbiota. Food Funct.

[36] Dai Z, Coker O O, Nakatsu G (2018). Multi-cohort analysis of colorectal cancer metagenome identified altered bacteria across populations and universal bacterial markers. Microbiome.

[37] Parker B J, Wearsch P A, Veloo A (2020). The Genus Alistipes: Gut Bacteria with Emerging Implications to Inflammation, Cancer, and Mental Health. Front Immunol.

[38] Zhang Y, Wang X L, Zhou M (2018). Crosstalk between gut microbiota and Sirtuin-3 in colonic inflammation and tumorigenesis. Exp Mol Med.

[39] Wen X, Wang H G, Zhang M N (2021). Fecal microbiota transplantation ameliorates experimental colitis via gut microbiota and T-cell modulation. World J Gastroenterol.

[40] Koh G Y, Kane A V, Wu X (2020). Parabacteroides distasonis attenuates tumorigenesis, modulates inflammatory markers and promotes intestinal barrier integrity in azoxymethane-treated A/J mice. Carcinogenesis.

[41] Hu K, Liao X X, Wu X Y (2022). Effects of the Lipid Metabolites and the Gut Microbiota in ApoE(-/-) Mice on Atherosclerosis Co-Depression From the Microbiota-Gut-Brain Axis. Front Mol Biosci.

[42] Zhang N, Liu J, Chen Z (2022). Integrated Analysis of the Alterations in Gut Microbiota and Metabolites of Mice Induced After Long-Term Intervention with Different Antibiotics. Front Microbiol.

[43] Ryan P M, Patterson E, Carafa I (2020). Metformin and Dipeptidyl Peptidase-4 Inhibitor Differentially Modulate the Intestinal Microbiota and Plasma Metabolome of Metabolically Dysfunctional Mice. Can J Diabetes.

[44] Yang J, Wei H, Zhou Y (2022). High-Fat Diet Promotes Colorectal Tumorigenesis Through Modulating Gut Microbiota and Metabolites. Gastroenterology.

[45] Zhu Y, Wei Y L, Karras I (2022). Modulation of the gut microbiota and lipidomic profiles by black chokeberry (Aronia melanocarpa L.) polyphenols via the glycerophospholipid metabolism signaling pathway. Front Nutr.

[46] Du L, Wang Q, Ji S (2022). Metabolomic and Microbial Remodeling by Shanmei Capsule Improves Hyperlipidemia in High Fat Food-Induced Mice. Front Cell Infect Microbiol.

[47] López-Hernández Y, Lara-Ramírez E E, Salgado-Bustamante M (2019). Glycerophospholipid Metabolism Alterations in Patients with Type 2 Diabetes Mellitus and Tuberculosis Comorbidity. Arch Med Res.

[48] Yuan Z, Yang L, Zhang X (2020). Mechanism of Huang-lian-Jie-du decoction and its effective fraction in alleviating acute ulcerative colitis in mice: Regulating arachidonic acid metabolism and glycerophospholipid metabolism. J Ethnopharmacol.

[49] Shan S, Wu C, Shi J (2020). Inhibitory Effects of Peroxidase from Foxtail Millet Bran on Colitis-Associated Colorectal Carcinogenesis by the Blockage of Glycerophospholipid Metabolism. J Agric Food Chem.

[50] Lv J, Jia Y, Li J (2019). Gegen Qinlian decoction enhances the effect of PD-1 blockade in colorectal cancer with microsatellite stability by remodelling the gut microbiota and the tumour microenvironment. Cell Death Dis.

[51] Hong X, Wang G, Liu X (2022). Lipidomic biomarkers: Potential mediators of associations between urinary bisphenol A exposure and colorectal cancer. J Hazard Mater.

[52] Tang X, Wang W, Hong G (2021). Gut microbiota-mediated lysophosphatidylcholine generation promotes colitis in intestinal epithelium-specific Fut2 deficiency. J Biomed Sci.

[53] Kadosh E, Snir-Alkalay I, Venkatachalam A (2020). The gut microbiome switches mutant p53 from tumour-suppressive to oncogenic. Nature.

[54] Zhou C B, Zhou Y L, Fang J Y (2021). Gut Microbiota in Cancer Immune Response and Immunotherapy. Trends Cancer.

[55] Li Q, Cui Y, Xu B (2021). Main active components of Jiawei Gegen Qinlian decoction protects against ulcerative colitis under different dietary environments in a gut microbiota-dependent manner. Pharmacol Res.

