# Supplementary material for: Berberine inhibits high fat diet-associated colorectal cancer through modulation of the gut microbiota-mediated lysophosphatidylcholine
Source: Int J Biol Sci. 2023 Apr 9;19(7):2097–113. doi: 10.7150/ijbs.81824 (PMC10158016; doi:10.7150/ijbs.81824)
Supplement: Supplementary file 1 — Supplementary figure and tables. [file ijbsv19p2097s1.pdf]

Supplementary Table 1. Relative abundance of selected bacteria that have an effect on intestinal tumors after high-fat diet or BBR intervention

	ND group	HFD group	HFD+BBR group	ND vs HFD (*P* value)	HFD vs HFD+BBR (*P* value)
Escherichia-Shigella	0.022%	6.169%	1.707%	0.004	0.631
Clostridium_sensu_stricto_1	0.005%	8.998%	0.009%	0.003	0.003
Lachnospiraceae_NK4A136_group	7.010%	0.363%	0.013%	0.004	0.123
Prevotellaceae_UCG-001	1.081%	0.001%	0	0.003	0.317
Akkermansia	0.121%	1.895%	58.822%	0.098	0.004
Parabacteroides	0.865%	3.544%	9.118%	0.749	0.036
Alistipes	7.752%	2.071%	0.115%	0.055	0.025

Supplementary Table 2. The list shows that the differential metabolites involved in glycerophospholipid metabolism pathway between HFD and ND group, or HFD and HFD+BBR group.

ID	Metabolite	HFD vs ND			HFD vs HFD+BBR		
		Ratio	*P* value	VIP	Ratio	*P* value	VIP
neg-M89T77	Acetaldehyde	6.285	0.002	1.667	10.178	0.002	2.864
neg-M512T244	LysoPC 14:0	2.345	0.002	1.138	2.259	0.002	1.434
pos-M468T302	LysoPE 17:0	0.333	0.015	1.326	0.257	0.004	2.160
neg-M494T198	LysoPS 16:1	8.281	0.002	1.718	8.961	0.002	2.438
neg-M455T367	LysoPG 14:0	0.187	0.002	1.462	0.086	0.002	3.050
neg-M469T543_2	LysoPG 15:0	0.253	0.002	1.458	0.113	0.002	2.978

Supplementary Table 3. The list shows that the genes involved in cancer pathway were identified with |log2(FC)| >3 and adjust *P* <0.01, in HFD+BBR group mice versus HFD group mice.

Gene name	Full name	Log2(Fold Change)
Fgf20	Fibroblast growth factor 20	13.05997
Fgf3	Fibroblast growth factor 3	7.742018
Cdkn2a	Cyclin dependent kinase inhibitor 2A	7.593844
Adcy8	Adenylate cyclase 8	7.329777
Shh	Sonic hedgehog	7.104894
Wnt10a	Wingless-type MMTV integration site family, member 10A	6.636194
Wnt6	Wingless-type MMTV integration site family, member 6	6.254606
Wnt7a	Wingless-type MMTV integration site family, member 7A	5.484407
Fzd10	Frizzled class receptor 10	5.334529
Il23a	Interleukin 23, alpha subunit p19	5.248131
Adcy1	Adenylate cyclase 1	4.282616
Pgf	Placental growth factor	4.281786
Fgf23	Fibroblast growth factor 23	3.848256
Wnt7b	Wingless-type MMTV integration site family, member 7B	3.706996
Ptch2	Patched 2	3.545008
Rac3	Rac family small GTPase 3	3.529274
Axin2	Axin 2	3.478435
Wnt3	Wingless-type MMTV integration site family, member 3	3.356096
Rxrg	Retinoid X receptor gamma	3.409176
Esr2	Estrogen receptor 2 (beta)	4.631725

Supplementary Table 4. The list shows that the genes involved in pro-inflammatory response were identified with |log2(FC)| >3 and adjust *P* <0.01, in HFD+BBR group mice versus HFD group mice.

Gene name	Full name	Log2(Fold Change)
Cxcl5	chemokine (C-X-C motif) ligand 5	7.501011
Adcy8	Adenylate cyclase 8	7.329777
Lcn2	Lipocalin 2	5.842324
Cxcl2	Chemokine (C-X-C motif) ligand 2	5.764953
Mmp13	Matrix metallopeptidase 13	5.385431
Il23a	Interleukin 23, alpha subunit p19	5.248131
Cxcl3	Chemokine (C-X-C motif) ligand 3	5.152942
Ccl24	Chemokine (C-C motif) ligand 24	4.730089
Adcy1	Adenylate cyclase 1	4.282616
S100a9	S100 calcium binding protein A9	4.220649
S100a8	S100 calcium binding protein A8	3.572487
Rac3	Rac family small GTPase 3	3.529274
Ccl7	Chemokine (C-C motif) ligand 7	3.496485
Cxcl1	Chemokine (C-X-C motif) ligand 1	3.467169
Ccl21d	Chemokine (C-C motif) ligand 21D	3.037678
Cxcl10	Chemokine (C-X-C motif) ligand 10	3.109878
Rxrg	Retinoid X receptor gamma	3.409176

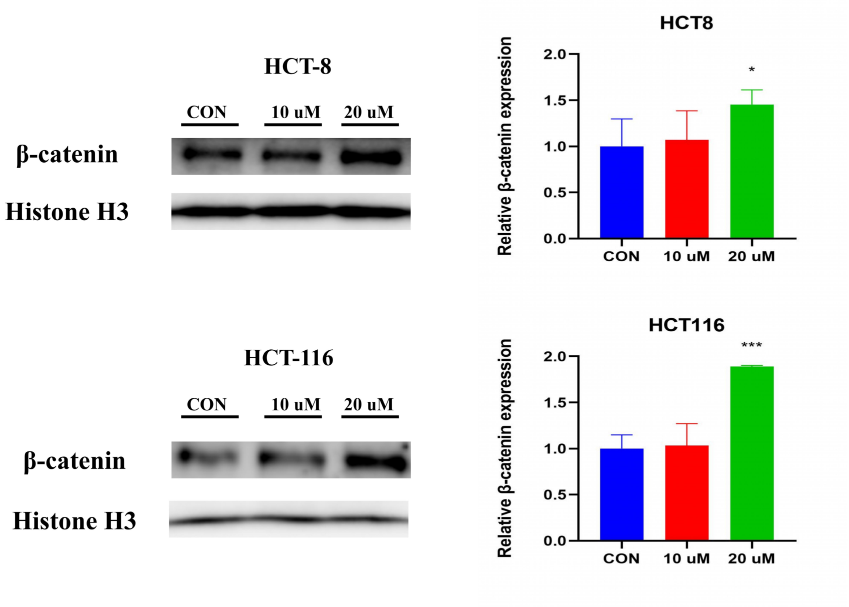

Fig S1. The BBR-associated differential metabolite LPC promotes β-catenin expression in the nucleus. Expression of nuclear β-catenin in HCT 8 cells and HCT 116 cells with or without LPC treatment.
